# Adherence to Antiretroviral Therapy in Jinja, Uganda: A Six-Year Follow-Up Study

**DOI:** 10.1371/journal.pone.0078243

**Published:** 2013-10-11

**Authors:** Martin Mbonye, Janet Seeley, Fatuma Ssembajja, Josephine Birungi, Shabbar Jaffar

**Affiliations:** 1 Social Science Programme, MRC/UVRI Uganda Research Unit on AIDS, Entebbe, Uganda; 2 School of International Development, University of East Anglia, Norwich, United Kingdom; 3 Department of Infectious Disease Epidemiology, London School of Hygiene and Tropical Medicine, London, United Kingdom; 4 The AIDS Support Organisation, Kampala, Uganda; Karolinska Institutet, Sweden

## Abstract

**Introduction:**

We report on the adherence experience of a group of people living with HIV on ART over six years in Uganda.

**Methods:**

Between 2005 and 2009, we followed up 41 participants who were also part of a clinical trial comparing home and facility based delivery of ART in Jinja, eastern Uganda. We conducted qualitative in-depth interviews at enrolment, 3, 6, 18 and 30 months to capture experiences with adherence over time. In 2011 we returned to these participants to find out how they were fairing with long term adherence. We managed to retrace 24 participants and interviewed them about their experience. We thematically analysed the data and compared findings over time.

**Results:**

Initially there were few barriers to adherence and many followed the adherence guidance closely. By year six, relaxation of these rules was noticeable although self-reported adherence continued to be high. Alcohol consumption was more common than before. Some relatives of the participants who had died claimed that some deaths were a result of alcohol. While participants reported that ART had allowed them to reclaim independence and return to work the changes in work and social routines created new challenges for adherence. Side effects like lipodystrophy were not only causing some stigma but for some tested their faith in the drugs. Many participants reported resumption of sexual lives but apart from those who selected same status partners, disclosure to new partners was minimal.

**Conclusion:**

Good adherence practice to ART wanes over the long-term, and people who may have disclosed at initiation find it difficult to do so to new partners once they are healthy. Further adherence interventions and support with disclosure over the course of therapy may need to be considered. (Words: 283)

## Introduction

About 25 million people are living with HIV-infection in Africa, of whom approximately seven million are on antiretroviral therapy [1]. Various studies, including systematic reviews, have described the challenges of retaining patients on antiretroviral therapy programmes but the evidence-base on adherence to therapy remains weak [2-6]. As moves to roll-out `treatment as prevention’ gather pace, there is an urgent need to understand and address, barriers of access to care and adherence to treatment. As Dieffenbach and Fauci [7] observed, major barriers exist in many places and the effectiveness of antiretroviral therapy will be compromised if those enrolled on to treatment are not retained on care [8]. Good adherence is not only essential to maintain patient health but also to prevent the spread of resistance to antiretroviral therapy. A systematic review published in 2006 suggested that adherence, measured using quantitative indicators, was considerably better in Africa than in the United States [9]. However, this seems implausible since retention in care is poor and mortality rates among those on antiretroviral therapy are considerably higher in Africa than in developed countries [10]. Given the complexity of measuring behaviour, further assessment of adherence is essential. 

The meaning of being infected with HIV has changed for patients over time from the certainty of imminent death and dying during the earlier part of the epidemic [11,12] to more recently of living with HIV as a chronic illness [12,13]. How this changed understanding of HIV-infection affects adherence to antiretroviral therapy is not clear. 

A number of studies have reported barriers to good adherence to antiretroviral therapy in the initial period after enrolment on to care, which have included: costs of visiting clinics to access care [14], which can be as high as 10-20% of monthly salary/wages per visit [15,16], occurrence of unpleasant side effects, such as lipodystrophy [17-19] and HIV-associated stigma [20,21]. 

Understanding of the challenges of adherence over time in Africa remains largely unknown. We report the challenges to adherence faced by patients after six years on antiretroviral therapy in Jinja, Uganda. 

## Methods

The study participants were part of a 5-year cluster-randomised trial conducted with The AIDS Support Organisation (TASO) in Jinja, Uganda between 2005 and 2009. The trial compared the effectiveness of two different delivery models: home-based care with trained lay delivering drugs to the home and providing adherence support versus providing standard facility-based care [15]. The trial showed that both strategies were approximately equally effective in terms of virologic response and mortality. The home-based care strategy was slightly cheaper for the health services and markedly cost-saving for participants. Recruitment into the trial began in 2005 and lasted for just under 2 years. Follow-up continued until January 2009. 

Nested within the trial was a social science study which selected and followed 41 of the trial participants. These participants were enrolled consecutively between October 2005 and April 2006 and stratified to ensure an equal number of participants by gender, trial arm and clinical/immunological stage [22]. Between the commencement of ART and the end of the trial in 2009 seven of the 41 (17%) participants had died.

After 2009, the participants were reintegrated into the delivery system of TASO which was a mixture of the facility, home and community based models of ART delivery. The facility-based model consisted of drug delivery at a designated TASO clinic. The community model also referred to as Community Drug Distribution Point (CDDP Model) involved ART delivery at a distribution point in the community to ease transport concerns of the people [23]. The distribution point may be a church, school compound or a local health centre within the community chosen by the participants. After initiation onto ART in the facility based model, the participants were evaluated clinically and psychosocially to determine their readiness to move to the CDDP model, a period that usually lasted between 3 to 6 months. Participants in the CDDP received one to two monthly ART refills by a lay trained health worker who was also charged with providing adherence counselling and monitoring for side effects.

Between May and July 2011 we conducted follow up visits to the participants in the qualitative study to find out about their lives after the end of the trial. We attempted to revisit the 34 people who were last known to be alive in January 2009. One of the interviewers who had been involved in the original qualitative study carried out these follow up visits.

This research was approved by the Science and Ethics Committee of The Uganda National Council of Science and Technology, The Uganda National Council for Science and Technology and the ethics committee of the London School of Hygiene and Tropical Medicine. All participants provided written informed consent.

## Results

The interviewer traced and interviewed 24 (12 men and 12 women) out of the 34 participants. Of the other 10, six had died sometime after the end of the trial and four had moved away and could not be contacted. Only 4/24 (17%) people (all women) reported not currently being in a relationship compared to 29/41 (71%) at enrolment in 2005. The median age of participants was now 46 (range 26 to 68) years. Only 4/24 (17%) people (all men) had acquired stable salaried employment, while others had informal employment as construction workers, petty traders, market vendors and farmers. Overall, 6 of the 24 (25%) participants had had one or more children since 2009. One man had three children born from two partners. One woman was pregnant at the time of interview in 2011, while another woman reported having miscarried 2 times in the recent past. 

The 6 participants who were reported to have died since 2009 comprised 4 men and 2 women. Relatives mentioned alcohol-related problems in the case of the three men. The other man and the two women were said to have died of malaria (a term often used to mean a non-specific fever). Four of the 6 who had died had reported to be adhering well to their treatment in January 2009 when last interviewed at end of the main trial.

In the next section we document the adherence experience of the 24 people who were still alive.

### Perceptions about Adherence Instructions

We found that most of the participants had become less vigilant in managing their infection than they had been in 2009. For example, some mentioned that they did not observe any immediate consequences of not following the instructions they had been given for their drug-taking regime when they began ART 1 participant described one such incident:

*I missed taking* [ARV drugs] *for three days. I had gone for a burial and I had packed some drugs hoping to spend two or three days but I found myself staying there for a week. At first I thought that the rules were so strict and I was asking myself how I could manage following them as I was a poor lady…Now I am used to the drugs I don’t mind whether I eat something first or not, I just take my drugs and I don’t have any problem* (Jane, aged 45)

Some of the instructions that they had been given as part of the psycho-social readiness counselling before they started taking drugs in 2005, included no smoking, no taking alcohol and strict observance of time of drug taking. Most of the participants told us that they were worried if the treatment was taken beyond 30 minutes late. By 2011, some participants admitted that they were not following all the instructions given and freely said this in interviews. One reported that joining friends and sharing alcohol was in itself a testament of being fully recovered, accepted and integrated into the community without worrying about the potential consequences on adherence:

… *I tell you, we have a drinking place of malwa* [Local beer] *but we share the local straws used for drinking that alcohol. You know it is kept in one pot and we can gather more than six people around that pot but we share the tube used to drink it. I also take some alcohol, and I can find a friend in a bar and we can share one bottle of beer*. (Henry, aged 47)

Alcohol consumption was also mentioned as being partly to blame for the deaths of some participants. We found in 2011 that consumption of alcohol was on the increase in this population among both men and women, even when those consuming it identified non consumption of alcohol as one of the key rules that they should follow. Most of the men had resumed the regular consumption of alcohol. One of the participants (Ssebunya, aged 49) was reportedly taking so much alcohol that it seemed to affect his livelihood and sometimes TASO staff found it hard to trace him for his drug supply. This is captured in the following observations by the interviewer who had noticed physical changes in the appearance of the participant:

Ssebunya had changed a lot, he had lost weight, he had dark skin. His cheeks were swollen, his fingers were shaking and his eyes were red as if he had had a sleepless night. As soon as he entered the room, I smelt alcohol and whenever he was talking I could smell alcohol, *I suspected* that he had drunk some alcohol the night before. When he started selling tomatoes and cabbages he got some money then he started to take alcohol. He started drinking heavily. He has friends with the same interests, they spend evenings in drinking places and one day a counselor from TASO went searching for him and he also found him in a drinking place. He took alcohol up to the extent of spending nights in drinking places and he is also a very keen smoker. 

### Work and Adherence

We had observed in 2009 after a median of 4 years since ART initiation, that as their health had improved some participants were taking up employment. By 2011 only 5 of 24 (21%) had relatively stable salaried jobs with one crediting ART for allowing him to `stabilize’, return to school and secure well-paid work. However the majority kept complaining about poverty and an inability to earn a secure income while managing the demands of adherence. Some admitted to swallowing antiretroviral drugs without food, as mentioned above, because they could not eat while working. Many of the other participants reported that they were struggling to juggle new work routines with proper adherence. 

*There are times when I miss the time. As I have told you that sometimes we spend nights on the lake* [fishing]*, when we come back on the lake shore in the morning, we take breakfast and go to sleep and for me my usual time of taking my drugs is 9.00 am and same time* [9.00pm] *in the evening. By that time I might be asleep and by the time I wake up it is already past my normal time…I can miss three times in a week*. (Ssebunya, aged 49)

Another participant who had been receiving drugs near the lake shore close to her business was affected when the decision was made to shift drug distribution to another point. The change affected the smooth running of her work because of the new location being more distant and she could not always afford the transport to get her drugs:

*I was getting my drugs from home and that was so easy for me. Now I have to walk to the distribution centre and at times I leave my fish out [to dry*]* and the wild birds eat it because I am not around. We spend a whole day there and when I try to leave my fish inside the house, it gets spoilt*. (Mary, aged 34)

### Continuing side effects

The side-effects of drugs, or worries about the potential side-effects in the future were still a concern to many. Some participants reported fat loss with sunken features around the face, a characteristic of lipodystrophy: six of the 24 (25%) participants, (three women and three men), expressed considerable discomfort about their appearance. One woman complained that her skin had darkened; two men and a woman mentioned that their finger and toe nails had become visibly dark and brittle. 

Drug-regimes were reported to have been changed in response to some of these side-effects, but for some the side-effects persisted and those affected had become resigned to the change in their appearance. A few of the affected people were beginning to question the effectiveness of ART:

*They told us that if you take these drugs, you don’t get other sicknesses but now you see how my eye looks like, I hoped that if I take the drugs for a long time all the sicknesses would go and I remain with only the virus* (Mary aged 34)**.**

However, others who had deformities because of ART or other visible signs which they had attributed to ART were beginning to feel uncomfortable with the way they looked. They had hoped that after 6 years on ART all would be well but the persistence of these signs brought about feeling of worry that gains were being reversed. Michael (aged 36) observed: 

…*You see it has changed its shape* [referring to the shape of mouth] *and even my face is pale with sunken eyes. Even though I try to add some jelly on my face it doesn’t change. People can think that I don’t bathe properly according to the way my skin looks. As a human being, I feel bad but there is nothing to do about it.*

Another female participant who was struggling with the side effects also seemed to have less confidence in the effectiveness of ART:

*I can’t say that they have worked well because as you see now my face it has changed from its normal shape, I don’t know whether it is a type of drug or it is because I have spent some time on them. I take Septrin and Efaviren*. (Agatha, aged 47)

### Experience with missed doses

Although few participants reported missing drugs (only three admitted to this), the good adherence planning reported during the 2005-2009 trial period had lapsed with drug taking becoming more erratic. However, almost all of participants reported that while they did not take their drugs `on time’, they only delayed swallowing drugs occasionally with some regularly going beyond the one hour limit that many suggested was allowable. It appeared that perceptions were shifting from strict time keeping to merely swallowing tablets. A case in point is participant Jane (aged 45) who commented that she would sometimes miss her drugs for a number of days and even when she took them, she was not always sure of the time: 

*…I don’t have an answer […] I just find myself missing time. I just estimate it as I don’t have a watch and a radio in my home. I used to know the time from the neighbour’s radio but we got misunderstandings and I no longer go to their home.*

Barriers to adherence did not only originate from the participants’ side, in one case, a participant blamed the provider: 

*I am no longer getting my medicine refills from the centre. I get them from the distribution point which is near here at the health centre. There was one day when they brought for me the type of drugs which I was not taking before. The nurse told me that the field officer was going to bring me the usual type of drugs on the next day. I waited for the whole day and I didn*’*t see the field officer. On the next day, I decided to go to the centre to pick my drugs. I missed one full day and one extra morning dose*. (Angela aged 54)

### HIV status disclosure after initiating ART

While most of the men and women said that they were comfortable disclosing their HIV status to family members, friends and workmates, as well as potential sexual partners, others felt that telling people about their status was now unnecessary. Some felt that they needed to be careful to keep their status a secret to avoid discrimination at work. Some feared that the negative public attitude may affect their livelihood: 

*You know if people know your HIV status, they can abandon your kiosk, now I am dealing in fresh foods, some people can stop buying them. Some people are still ignorant, one can think that if a person with HIV touches a mango the virus can be spread to him or her* (Michael aged 36)

There seemed to be little motivation to continue disclosing to new people who had never known that they were HIV-positive. Jonathan (aged 67) felt that it was not necessary for him to disclose his status any more, given that he looked healthy. However his desire for secrecy was causing him some problems because the dosage time he had been given was not working for him, due to work commitments, and he was in the process of asking the counsellor to change it. He was struggling to adhere to his drugs: 

*There is no reason why I should inform them* [new people including employer]*, they are not going to help me in anything. Lucky enough my wife knows and she is the one looking after me*. 

### Experiences with sexual relationships

Attitudes to sex and pregnancy had already begun to change in 2009, when the main study ended. This change had persisted with most of those who had vowed to abstain from sex when they had begun ART actively engaged in sex with new partners. Disclosure to such partners was proving to be a challenge. Of the 10 participants who had got new partners, five were unsure of their partner’s status. These 5 had not disclosed their own status to that new partner. 

Seven of these10 participants reported having had a child or a pregnancy in the last three years. Some did know their new partners status: 

”*I found myself having resumed sex issues again. I never expected to look like how I am now. I never expected to get a sexual partner…We met at our treatment place and we got attracted to each other….We both have the disease* [HIV/AIDS] *and we had agreed on using condoms. I don’t know what happened to my friend, only to find myself pregnant. She (baby*)* is eight months old….For me I never liked to get a child but I just saw that I was pregnant and you never know I might say that I will not get a second child and I find myself getting one*”. (Matilda, aged 40)

Having new sexual partners potentially posed questions for the participant because it involved worrying about the pregnancy outcome and perhaps the need to change drugs at some point because of changes brought by pregnancy.

There appeared to be a preference for partners who were also HIV-positive. Sero-sorting it was argued allowed them to avoid worrying about infecting someone else. The new partner, who would be taking drugs themselves, would help with proper adherence:

*I no longer want to infect others, and a person who is also HIV positive knows* [the knowledge of what HIV positive people experience] *how they are expected to behave like using condoms and drug taking*. (William, aged 45)

## Discussion

Our findings show that the incorporation of an HIV identity does not follow a linear process as has been suggested [24]. Joining a support organisation has been documented as a turning point [25] with the expectation that such organisations do provide adequate support to PLHIV. Our study population was part of The AIDS Support Organisation which has historically emphasised positive living including the public embrace of status through the time tested positive living approaches [26]. Almost all TASO clients are given counselling support to disclose their HIV status and disclosure rates at the time of ART initiation were close to 100% in this population. 

The importance of adherence was well understood by our study participants and they managed to follow the ‘strict’ instructions in the first few years after initiating ART aided by the strong psychosocial support system [27,28]. Such efforts may have contributed to the high adherence rates that have been reported in much of Sub-Saharan Africa [10]. However with improvements in health comes multiple and sometimes unanticipated adherence challenges. For example our participants who had resumed sexual relationships often failed to disclose their HIV sero status to new sexual partners as a strategy to secure a stable source of livelihood, especially the women. Such relationships inevitably led to pregnancies/child birth and the non-disclosure presented challenges for proper adherence. It has already been documented that PLHIV tend to reclaim sexual and reproductive lives after starting ART [29,30] and that pregnant women are particularly vulnerable to poor adherence especially in the first three months; a challenge our participants would relate to [31]. Non-disclosure especially to close companions like sexual partners has been associated with poor adherence [2,9]. Our finding, that HIV disclosure becomes a challenge some years after ART initiation, suggests that people on ART may need continued follow-up support to disclose their status to new partners, similar to the support provided when ART is initiated.

It is worth noting the relaxation of the rules of adherence from the time of the initiation of ART. At the start of treatment, all our participants had vowed strictly to adhere to instructions which included: no smoking, no alcohol, reduction in number of partners and having food available to take with the drugs [27]. This was no longer the case five years later. Probably most concerning was that alcohol consumption was reported to be high for some and yet it is associated strongly with poor adherence [32]. Our findings also demonstrate the difficulty in managing an HIV identity in the context of a suspicious and stigmatizing community. Some participants opted to hide their status in the work context fearing that public knowledge could lead to loss of income. Similar reactions about the challenges of taking ART and building a life with HIV have been identified in other settings [33]. Others had developed side effects which were causing some discomfort and reduction in the faith in medication they had built over the years. Adherence messages may need to be enforced periodically, particularly in relation to the avoidance of alcohol consumption and safer sex and that given the persistence of side effects, regimens with poor toxicity profiles, such as stavudine, should be completely phased out. Some of our study population reported having regimens substituted but there were no reports of failed therapy. It is important to note that our study population were clients of an NGO which believes strongly in support, counselling and empowerment of patients and at the time of ART initiation, these clients are likely to have been among the most knowledgeable about ART and the need to adhere in Africa. The need for regular periodic intervention to sustain high levels of adherence in patients over the long term is likely to be higher in other populations.

## Conclusion

These findings highlight a number of issues that affect adherence and potentially affect long-term survival outcomes. While ART opens new social and economic opportunities, after regaining better health, PLHIV have to think about fitting in without compromising individual identities and values. Long term adherence interventions that focus on unique individual situations should be considered. These might include: regularly re-engaging with PLHIV to understand and react to changing contexts, re-emphasising the rules of adherence by focusing on specific individual needs and challenging any complacency that might grow into poor adherence. Larger studies among long term users of ART might offer greater insight into the long term challenges and opportunities among populations such as ours. 
